# NOD-Like Receptor P3 Inflammasome Controls Protective Th1/Th17 Immunity against Pulmonary Paracoccidioidomycosis

**DOI:** 10.3389/fimmu.2017.00786

**Published:** 2017-07-10

**Authors:** Claudia Feriotti, Eliseu Frank de Araújo, Flavio Vieira Loures, Tania Alves da Costa, Nayane Alves de Lima Galdino, Dario Simões Zamboni, Vera Lucia Garcia Calich

**Affiliations:** ^1^Department of Immunology, University of São Paulo, São Paulo, Brazil; ^2^Department of Cell Biology, School of Medicine of Ribeirão Preto, University of São Paulo, São Paulo, Brazil

**Keywords:** pulmonary paracoccidioidomycosis, NOD-like receptor P3 inflammasome, Th1/Th17 immunity, regulatory T cells, immunoregulation

## Abstract

The NOD-like receptor P3 (NLRP3) inflammasome is an intracellular multimeric complex that triggers the activation of inflammatory caspases and the maturation of IL-1β and IL-18, important cytokines for the innate immune response against pathogens. The functional NLRP3 inflammasome complex consists of NLRP3, the adaptor protein apoptosis-associated speck-like protein, and caspase-1. Various molecular mechanisms were associated with NLRP3 activation including the presence of extracellular ATP, recognized by the cell surface P2X7 receptor (P2X7R). Several pattern recognition receptors on innate immune cells recognize *Paracoccidioides brasiliensis* components resulting in diverse responses that influence adaptive immunity and disease outcome. However, the role of NLRP3 inflammasome was scantily investigated in pulmonary paracoccidioidomycosis (PCM), leading us to use an intratracheal (i.t.) model of infection to study the influence of this receptor in anti-fungal immunity and severity of infection. For *in vivo* studies, *C57BL/6* mice deficient for several NLRP3 inflammasome components (*Nlrp3*^−/−^, *Casp1/11*^−/−^, *Asc*^−/−^) as well as deficient for ATP receptor (*P2x7r*^−/−^) were infected *via* i.t. with *P. brasiliensis* and several parameters of immunity and disease severity analyzed at the acute and chronic periods of infection. Pulmonary PCM was more severe in *Nlrp3*^−/−^, *Casp1/11*^−/−^, *Asc*^−/−^, and *P2x7r*^−/−^ mice as demonstrated by the increased fungal burdens, mortality rates and tissue pathology developed. The more severe disease developed by NLRP3, ASC, and Caspase-1/11 deficient mice was associated with decreased production of IL-1β and IL-18 and reduced inflammatory reactions mediated by PMN leukocytes and activated CD4^+^ and CD8^+^ T cells. The decreased T cell immunity was concomitant with increased expansion of CD4^+^CD25^+^Foxp3 regulatory T (Treg) cells. Characterization of intracellular cytokines showed a persistent reduction of CD4^+^ and CD8^+^ T cells expressing IFN-γ and IL-17 whereas those producing IL-4 and TGF-β appeared in increased frequencies. Histopathological studies showed that all deficient mouse strains developed more severe lesions containing elevated numbers of budding yeast cells resulting in increased mortality rates. Altogether, these findings led us to conclude that the activation of the NLRP3 inflammasome has a crucial role in the immunoprotection against pulmonary PCM by promoting the expansion of Th1/Th17 immunity and reducing the suppressive control mediated by Treg cells.

## Introduction

*Paracoccidioides brasiliensis* is the etiological agent of paracoccidioidomycosis (PCM), the most prevalent systemic mycosis in Latin America (1). The pattern recognition receptors (PRRs) expressed on phagocytic cells recognize pathogen-associated molecular patterns (PAMPs) on *P. brasiliensis* surface and regulate the innate and adaptive phases of immunity. The different signaling pathways used by these PRRs to activate immune cells result in different patterns of cell activation, production of diverse pools of cytokines and chemokines triggering diverse immune mechanisms. In human PCM and experimental models, the prevalent expansion of Th1/Th17 cells with reduced expansion of Treg cells characterize the benign disease, whereas elevated proliferation of Th2/Th9 and Treg cells is associated with severe PCM (2–11).

The toll-like receptors (TLRs) play a crucial role in the recognition of fungal pathogens such as *P. brasiliensis, Candida albicans, Aspergillus fumigatus, Cryptococcus neoformans*, and others (8, 12–16). TLR2 and TLR4 sense *P. brasiliensis* mediated by MyD88-dependent signaling that leads to cytokines production by alveolar and peritoneal macrophages (6, 7, 17, 18). These components were also seen to control the adaptive immunity and severity of disease developed by *P. brasiliensis* infected mice (6, 7, 18). In addition, C-type lectin receptors (CLRs) like mannose receptor (MR) and dectin-1 as well as the complement receptors 3 play an important role in *P. brasiliensis* recognition and activation of immune cells (8, 19–22). Altogether, these studies have demonstrated that in pulmonary PCM, as in other infectious pathologies, PRRs are key elements that govern protective immunity, which must be tightly controlled by anti-inflammatory mechanisms as those mediated by Treg cells and their products (5–7).

The NOD-like receptor P3 (NLRP3) belongs to the NOD family of cytosolic PRRs that detect intracellular PAMPs well as danger signals, named danger associated molecular patterns (DAMPs). Under cell activation, components of NLR family can aggregate into large cytoplasmic complexes called inflammasomes (23). The members of this family have a LRR (leucine-rich repeat) domain in the C-terminal structure, a nucleotide-binding and oligomerization domain containing a neuronal apoptosis inhibitory protein, and a N-terminal caspase recruitment domain (CARD) or pyrin (PYD) domain. In the canonical pathway of NLRP3 inflammasome activation, NLRP3 oligomerization is initiated through the apoptosis-associated speck-like protein (ASC) containing C-terminal CARD that is recruited to the complex *via* NLRP3-PYD-ASC-PYD interaction. ASC associates with procaspase-1 *via* CARD interaction, leading to caspase-1 activation that promotes the processing of pro-IL-1β and pro-IL-18 into their mature forms (24–26). The NLRP3 inflammasome is activated by a large repertoire of PAMPs and DAMPs, including ATP, uric acid crystals, silica, aluminum hydroxide, asbestos, reactive oxygen species (ROS), and bacterial or viral RNA (27, 28). Besides the canonical pathway, a non-canonical pathway that utilizes caspase-11 has been shown to activate NLRP3 inflammasome (29, 30). The non-canonical NLRP3 inflammasome can be directly activated by LPS derived from Gram-negative bacteria and by some fungi, which are delivered into the cytosol and activate caspase-11. This, in turn, triggers the opening of the pannexin-1 channel that induces the K + efflux required for NLRP3 inflammasome activation and release of mature IL-1β (30, 31). The first signal for pro-IL-1β processing is through pro-IL-1β and NLRP3 expression mediated by NF-kB transcription that is potentially activated by TLRs and CLRs, and the second signal is the proteolytic processing of pro-caspase-1 by activated NLRP3 (25). NLRP3 is the NLR mostly involved in the immunity against fungal infections (32–34). In *C. albicans* infection, the canonical and non-canonical pathways of NLRP3 inflammasome activation were shown to be essential for mediating IL-1β secretion (29, 35). *In vivo* studies of disseminated *C. albicans* infection showed that NLRP3, Syk, ASC, and caspase-1 are fundamental to control disease severity and regulate the adaptive antifungal immune response through the induction of Th1 and Th17 development (36, 37). In an invasive pulmonary model of aspergillosis, NLRP3 and AIM2 were required to engage the inflammasome to trigger innate immune responses against *A. fumigatus*; mice lacking both AIM2 and NLRP3, but not mice lacking a single inflammasome receptor, were hyper susceptible to invasive aspergillosis (38). The pioneer study of Tavares et al. demonstrated that following *P. brasiliensis* infection, the production of mature IL-1β by bone marrow-derived dendritic cells depends on NLRP3 and caspase-1 activation (39), although the fungal molecules associated with this process are still unknown. However, K + efflux, ROS generation, lysosomal acidification, and cathepsin B release to the cytosol were seen to be required to the NLRP3 inflammasome activation by *P. brasiliensis* (39). Furthermore, a recent work of our lab showed that a dectin-1-Syk mediated mechanism controls NLRP3 inflammasome activation by *P. brasiliensis* infected macrophages of resistant A/J mice (21). In another study using a murine model of systemic PCM induced by intravenous infection, the susceptibility of NLRP3 and caspase-1 knockout mice to *P. brasiliensis* infection was evaluated and their increased susceptibility was associated with reduced IL-18 production and Th1 immunity (40).

Because the human disease is thought to be acquired by the pulmonary route, and diverse routes of infection induce different patterns of immunity and disease severity (41), in the present study, we sought to investigate the role of NLRP3 inflammasome in the pulmonary infection caused by *P. brasiliensis*. Therefore, several C57BL/6 mouse strains deficient for NLRP3 inflammasome components (*Nlrp3*^−/−^, *Casp1/11*^−/−^, *Asc*^−/−^ mice) as well as deficient for the ATP receptor (*P2x7r*^−/−^ mice) were intratracheally infected with 1 × 10^6^ *P. brasiliensis* yeasts and compared with their wild type (WT) controls. Several parameters of pulmonary infection and immune response were evaluated at the acute and chronic phases of the disease. Our results indicated that NLRP3 inflammasome plays a crucial role in the control of the innate and adaptive immunity against *P. brasiliensis* infection. This control was associated with IL-1β and IL-18 secretion induced by caspase-1 activity starting at an early phase of infection. The activation of NLRP3 inflammasome was seen to be essential to control fungal growth and activate protective T cell immunity. This protective inflammatory response was composed of increased numbers of PMN leukocytes and activated CD4^+^ and CD8^+^ T lymphocytes that migrate to the site of infection. Furthermore, NLRP3 inhibited the expansion and migration of regulatory T (Treg) cells resulting in a well-balanced and protective Th1/Th17 immunity.

## Materials and Methods

### Mice

Wild type (*WT*), *Nlrp3*^−/−^, *Casp1/11*^−/−^, *P2x7r*^−/−^, and *Asc*^−/−^ *C57BL/6* mouse strains were obtained from our Isogenic Unit (Immunology Department of Institute of Biomedical Sciences of University of São Paulo, Brazil). *Casp1/11*^−/−^ mice (42) were provided by Dr. Flavell, R. (Howard Hughes Medical Institute, Yale University School of Medicine), *As*c^−/−^ mice (43) were provided by Dr. Zamboni, D. S. (University of São Paulo, Ribeirão Preto, São Paulo, Brazil), *Nlrp3*^−/−^ mice (44) and *P2x7r*^−/−^ (45) mice were obtained from Jackson Laboratories and used at 8–11 weeks of age. Specific pathogen free mice were fed with sterilized laboratory chow and water *ad libitum*. Animal experiments were performed in strict accordance with the Brazilian Federal Law 11,794 establishing procedures for the scientific use of animals, and the State Law establishing the Animal Protection Code of the State of São Paulo. All efforts were made to minimize suffering, and all animal procedures were approved by the Ethics Committee on Animal Experiments of the Institute of Biomedical Sciences of University of São Paulo (Proc.76/04/CEUA).

### Fungus

*Paracoccidioides brasiliensis* (Pb 18 strain), isolated from a young patient in 1929, was maintained by weekly subcultivation in semisolid Fava Netto’s medium at 35°C (46). Yeast cells were collected, washed, and adjusted to 20 × 10^6^ cells/mL. Viability was determined with Janus Green B vital dye (Merck Frankfurter Straße, Darmstadt, GER) and was always higher than 85%. The presence of LPS in all used solutions was determined by the Limulus amebocyte lysate chromogenic assay (Sigma-Aldrich, St. Louis, MO, USA) and always showed LPS levels <0.015 EU/mL.

### Intratracheal Fungal Infection

Mice were anesthetized and submitted to intratracheal (i.t.) *P. brasiliensis* infection as previously described (47). Briefly, after intraperitoneal injection of ketamine (90 mg/kg) and xylazine (10 mg/kg), animals were infected with 1 × 10^6^ Pb18 yeast cells, contained in 50 µL of PBS, by surgical i.t. inoculation, which allowed dispensing of the fungal cells directly into the lungs. The skin was then sutured, and mice were placed under a heat lamp until they recovered from anesthesia.

### Colony Forming Units (CFUs) Assays

To assess the viable number of CFU in target organs, lungs, and livers from *C57BL/6, Nlrp3*^−/−^, *Casp1/11*^−/−^, *P2x7*r^−/−^ and *Asc*^−/−^ mice were aseptically removed, weighted, and homogenized in 5 ml PBS using tissue grinders as previously described (48). In brief, 100 µL aliquots of 50- and 100-fold dilutions from organ homogenates were plated onto petri dishes containing brain heart infusion agar (Difco) supplemented with 5% *P. brasiliensis* 192 culture filtrate and 4% (v/v) horse serum (Instituto Butantan, São Paulo, Brazil) and incubated at 36°C. The fungal counting started at day 5 after plating, which is the period when the fungus starts to grow. Thereafter, the counting was performed daily and number of CFUs per gram of tissue determined.

### Mortality Rates

Mortality studies were done with groups of 8 *C57BL/6, Nlrp3*^−/−^, *Casp1/11*^−/−^, *P2xR7*^−/−^, and *Asc*^−/−^ mice inoculated i.t. with 1 × 10^6^ yeast cells. Deaths were registered daily and experiments were repeated twice.

### Histopathological Analysis

Histopathological analysis of lungs and livers was performed as previously described (49). Briefly, lungs and livers from *C57BL/6, Nlrp3*^−/−^, *Casp1/11*^−/−^, *P2x7r*^−/−^, and *Asc*^−/−^ mice were fixed in formalin (10%) and embedded in paraffin. Tissues sections were stained with hematoxylin–eosin (H&E) for analysis of the lesions and Grocott for fungal evaluation. Morphometrical analysis was performed using Nikon DXM 1200c digital camera and Nikon NIS Elements AR 2.30 software. Results were expressed as the mean ± SD for the total area of lesions.

### Cytokines Detection

The levels of IL-1β, IL-18, TNF-α, and IL-6 were measured in lung homogenates by ELISA with antibodies pairs purchased from eBioscience according to the manufacturer’s protocol. The absorbance values were determined using a spectrophotometric plate reader (VersaMax, Molecular Devices).

### Flow Cytometry Analysis

Cells obtained from lung homogenates at 48 h, 2, 4, and 10 weeks after i.t. infection were isolated as previously described (50). In brief, the lungs were excised, minced, and digested with collagenase (1 mg/ml, Sigma) and DNase (30 µg/mL, Sigma) in RPMI buffer (5% fetal calf serum, Sigma). Leukocytes were isolated with Percoll (20%, Sigma). The numbers of leukocytes were adjusted to 1 × 10^6^ cells/mL and suspended in staining buffer (PBS, 2% fetal calf serum and 0.1% NaN3). Fc receptors were blocked using unlabeled anti-CD16/32 antibodies (1 µg/ml; BD Biosciences), and cells were stained for 20 min at 4°C with the monoclonal antibodies diluted at optimal concentration: pacific blue (PB)-labeled anti-CD45, anti-CD25, anti-IL-1β, anti-F4/80 (5 µg/ml); phycoerythrin (PE)-labeled anti-CD4, anti-Ly6G, anti-Syk kinase (2 µg/ml); PECy7-labeled anti-CD8, anti-CD4, anti-IL-4 (2 µg/ml); allophycocyanin (APC)-labeled anti-CD44, anti-FoxP3, anti-CD8, anti-CD86, anti-IFN-γ (5 µg/ml); APC-Cy7-labeled, anti-CD45 (2 µg/ml); Brilliant Violet 510 (BV)-labeled anti-IL-17 (2 µg/ml); peridinin chlorophyll protein (PerCP)-labeled anti-TGF-β, anti-CD62L, anti-MHCII (2 µg/ml); fluorescein isothiocyanate-labeled anti-CD11b, anti-CD3 (5 µg/ml); (from BD Biosciences or BioLegend). Cells were washed twice with staining buffer, fixed with 1% paraformaldehyde (Sigma), and acquired using a FACSCanto II equipment and software FlowJo (Tree-Star).

### Intracellular Cytokines Measurement

The intracellular detection of cytokines was performed as previously described (49). Briefly, cells were labeled for surface molecules with monoclonal antibodies: anti-CD45, anti-CD8 (PB), anti-CD4 (PE), and anti-CD25 (PB) (BD Biosciences). For intracellular staining, cells were treated with Cytofix/Cytoperm kit (BD Biosciences), according to manufacturer’s protocol and labeled with anti-IL-1β (PECY7), anti-IFN-γ (APC), anti-IL-17 (BV), anti-IL-4 (PECy7), anti-TGF-β (PerCP) monoclonal antibodies. For Treg characterization, plots were gated on CD4^+^CD25^+^ cells. Cells were fixed with 1% paraformaldehyde (Sigma) and acquired at 50,000 events using a FACSCanto II equipment and FACSDiva software (BD Biosciences) using software FlowJo (Tree-Star).

### RNA Isolation and cDNA Synthesis

Lungs were homogenized in TRIzol reagent using tissue grinders. RNA isolation and purification was performed as described (49) using Ultraclean Tissue & Cells RNA Isolation Kit (MO BIO Laboratories) according to the manufacturer’s protocol. RNA concentration was assessed on a NanoDrop ND-1000 spectrophotometer. c-DNA synthesis was performed using the High Capacity RNA-to-cDNA kit (Applied Biosystems) following the manufacturer’s instructions.

### Real-time Quantitative Polymerase Chain Reaction

The cDNA was amplified using TaqMan Universal PCR Master Mix (Applied Biosystems) according to manufacturer’s protocol. The primers used were: IL-1β Mm01336189_m1; IL-18 Mm00434225_m1; ASC (Pycard) Mm00445747_ g1; Syk (Sykb) Mm01333032_m1; Casp-1 Mm00438023_m1; Nlrp3 Mm00840904_m1 (Applied Biosystems). Data were normalized to GAPDH gene expression. TaqMan PCR assays were performed on a MxP3000P QPCR System, and data were developed using the MxPro QPCR software (Stratagene). The average threshold cycle (CT) values of samples were normalized to CT value of GAPDH gene. The relative expression was determined by the 2^−ΔΔ^CT method.

### Statistics

Values represent means ± SD. Comparison among multiple groups was done with ANOVA non-pared test, and multiple comparisons according Bonferroni Differences between survivals were compared by log-rank test. All statistical analyses were performed using the software GraphPad Prism (GraphPad Software, Inc.).

## Results

### NLRP3 Inflammasome Controls the Pulmonary and Hepatic Fungal Loads of Mice i.t. Infected with *P. brasiliensis* Yeasts

**Figure 1:**
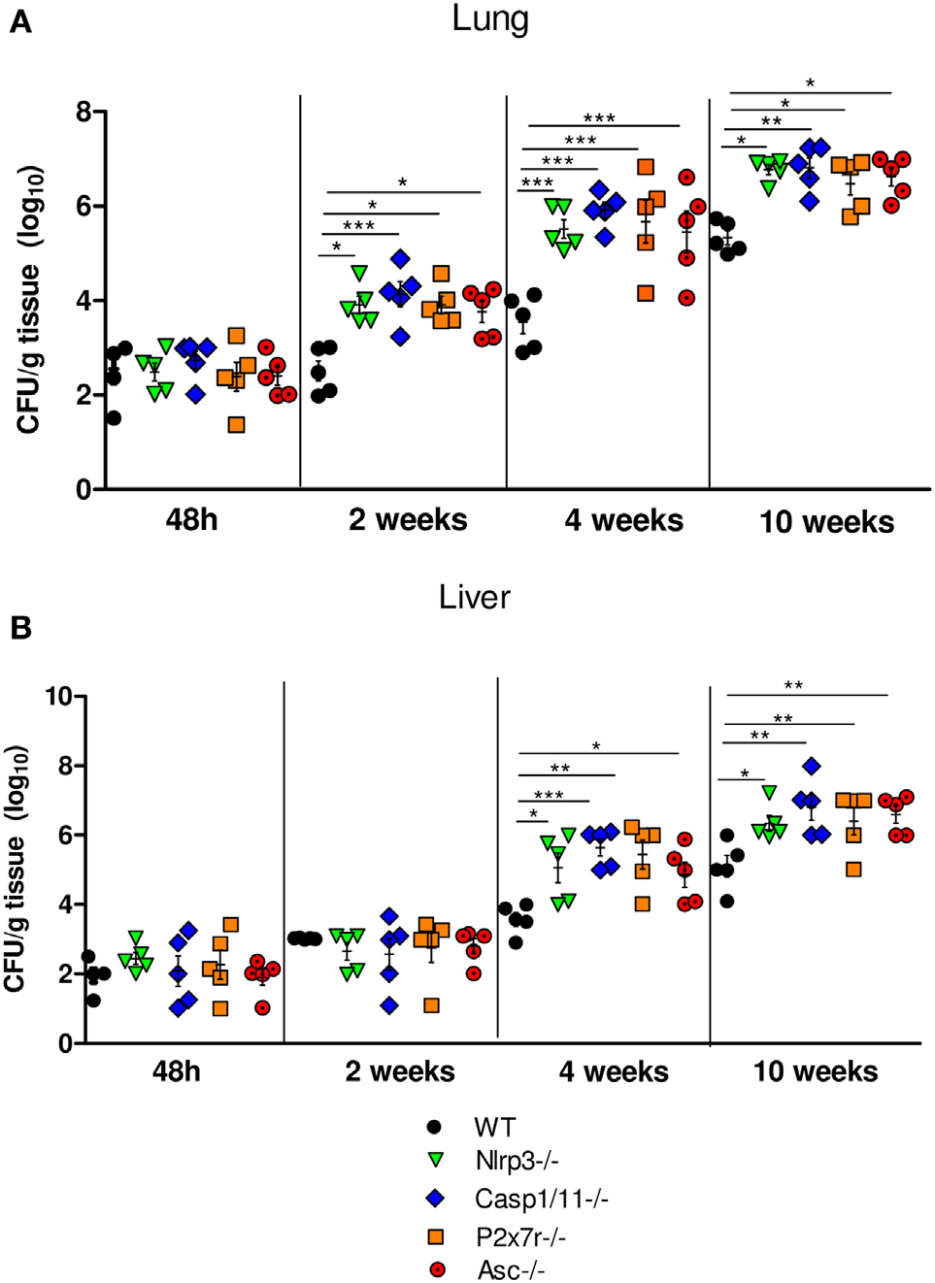
NOD-like receptor P3 (NLRP3) inflammasome controls the pulmonary and hepatic fungal loads of mice i.t. infected with *P. brasiliensis* yeasts. Disease severity was assessed by a CFU assay using macerated lungs **(A)** and livers **(B)** of *WT* mice, *Nlrp3*^−/−^, *Casp1/11*^−/−^, *P2x7r*, and *Asc*^−/−^ mice infected with 1 × 10^6^ yeasts cells *via* i.t. at 48 h, 2, 4, and 10 weeks of infection. Data are expressed as the number of CFU/g tissue of an experiment using five mice. Equivalent results were obtained in a second experiment using the same number of infected animals (*P < 0.05, **P < 0.01, and ***P < 0.001).

To evaluate the function of NLRP3 inflammasome in pulmonary PCM, we first investigated the control of fungal burdens by WT and NLRP3 inflammasome deficient (*Nlrp3*^−/−^, *Casp1/11*^−/−^, and *Asc*^−/−^) mice i.t. infected with (1 × 10^6^) yeasts cells. Mice deficient for the ATP receptor (*P2x7r*^−/−^) were also studied. Lung and liver of mice were excised, macerated, and the homogenates were diluted at 1:100 and plated in Petri dishes. The fungal counting started at day 5 after plating, which is the period when the fungus starts to grow. Thereafter, the counting was performed daily. The degree of infection was analyzed from the acute to the late phases (48 h, 2, 4, and 10 weeks) of infection. In the acute phase (48 h p.i.), no differences in the pulmonary and hepatic fungal loads between WT and NLRP3 deficient mice were seen. In contrast, increased fungal recovery at weeks 2, 4, and 10 of infection were observed in the lungs of *Nlrp3*^−/−^, *Casp1/11*^−/−^, *P2x7r*^−/−^, and *Asc*^−/−^ mice, that was more prominent at 10 weeks of infection (Figure 1A). At weeks 4 and 10 after infection increased, fungal burdens were seen in the liver of all deficient mice (Figure 1B). These data demonstrate that NLRP3 played an important role in the control of pulmonary fungal load and its dissemination to the liver.

### *P. brasiliensis* Infection Induces the Expression of mRNA from NLRP3-Inflammasome Related Components That Is Abolished in *Nlrp3*^−/−^, *Casp1/11*^−/−^, and *Asc*^−/−^ Mice

**Figure 2:**
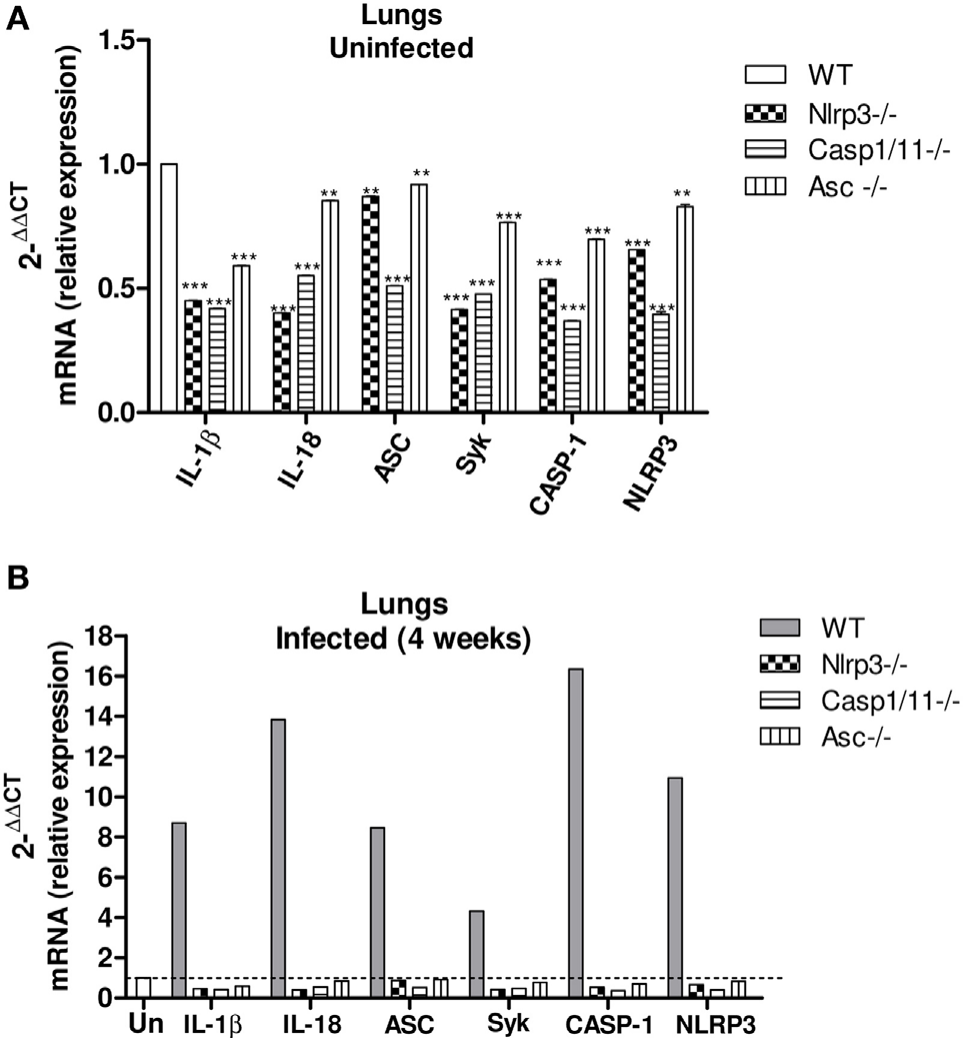
*Paracoccidioides brasiliensis* infection induces an increased expression of mRNA from NLRP3 inflammasome-related components, which is abolished in *Nlrp3*^−/−^, *Casp1/11*^−/−^, and *Asc*^−/−^ mice. Real-time quantitative polymerase chain reaction analysis of the IL-1β, IL-18, Asc, Casp-1, Nlrp3, and Syk genes expression in the lung macerates of *Nlrp3*^−/−^, *Casp1/11*^−/−^, *Asc*^−/−^, and WT uninfected mice **(A)** and at week 4 of infection **(B)**.

The activation of NLRP3 inflammasome requires two steps, the first mediated by PRRs and NFkB activation. Pathogen components, which are TLRs agonists as well as inflammatory cytokines such as TNF-α and IL-1β can trigger NFκB activation through diverse signaling pathways (51, 52). Therefore, caspase-1 can trigger NFkB activity *via* activity of pro-inflammatory cytokines produced by ASC-dependent inflammasome activation. Additionally, recent studies have shown that caspase-1 and ASC can direct NFkB activation, and this process is independent on the enzymatic activity of caspase-1 (52, 53). Indeed, caspase-1 can trigger TLR2- and TLR4-mediated NFκB activation *via* MyD88 adaptor like (MAL) for signal transduction (54). This led us to hypothesize that the absence of NLRP3 components might reduce the mRNA expression of cytokines and other NLRP3-related components. Therefore, the expression of IL-1β, IL-18, NLRP3, Caspase-1, ASC, and Syk mRNA was characterized in lung macerates of *WT, Nlrp3*^−/−^, *Casp1/11*^−/−^, and *Asc*^−/−^ mice at week 4 after infection. Increased expression of IL-1β, IL-18, NLRP3, Caspase-1, ASC, and Syk mRNA was detected in the lungs of WT mice compared to knockouts mice and uninfected mice as a control (Figures 2A,B) demonstrating that *P. brasiliensis* infection induces an increased synthesis of components associated with NLRP3 inflammasome. In addition, the increased expression of Syk mRNA suggests the contribution of this enzyme in the activation process of NLRP3 as shown in our previous study (21).

### Secretion of IL-1β and IL-18 but Not TNF-α and IL-6 Depends on NLRP3 Activation

**Figure 3:**
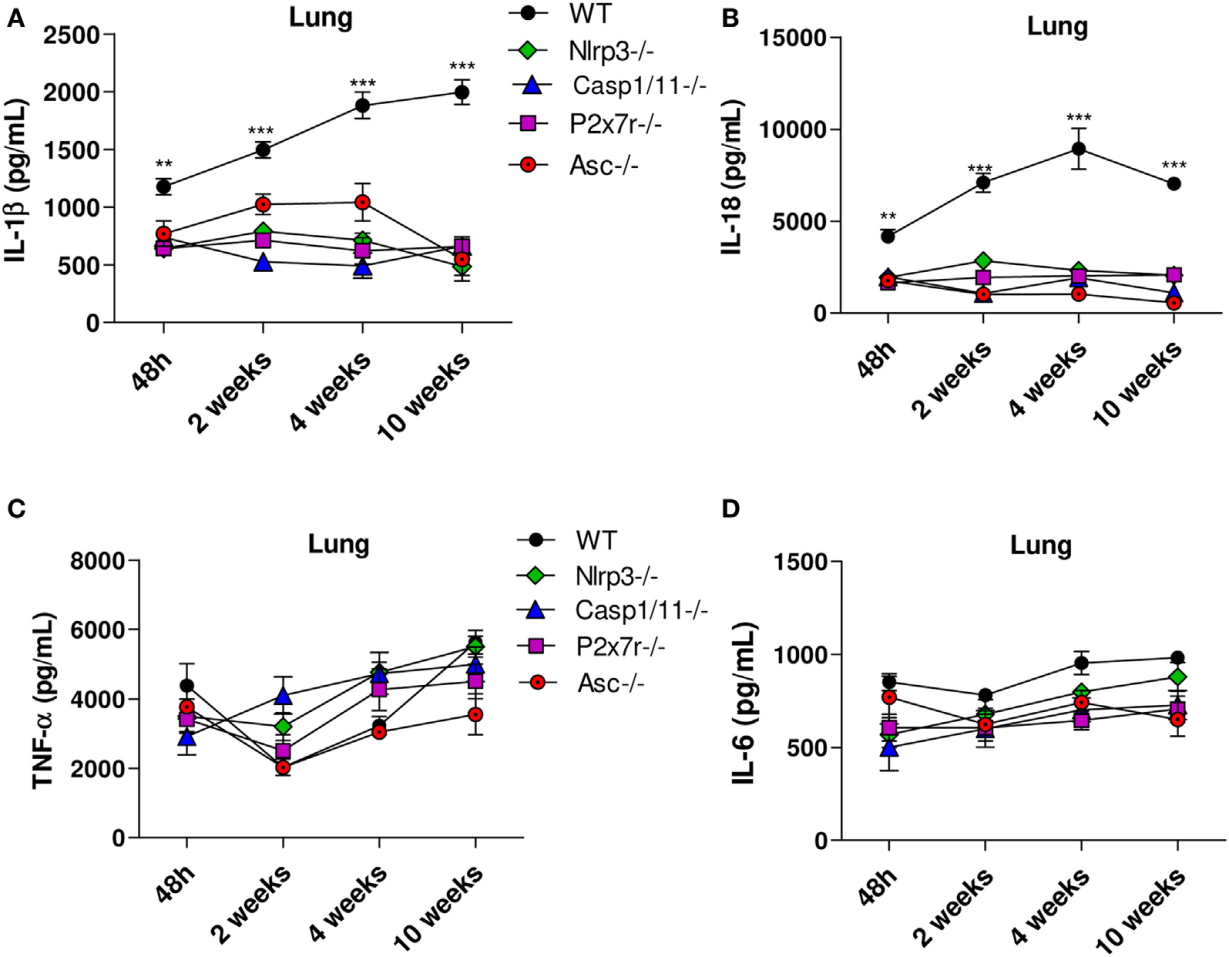
Secretion of IL-1β and IL-18 but not TNF-α and IL-6 is controlled by NLRP3 inflammasome activation. The supernatants of macerated lungs from *WT, Nlrp3*^−/−^, *Casp1*^−/−^ *P2x7r*^−/−^, and *Asc*^−/−^ mice infected *via* i.t. with 1 × 10^6^ *Paracoccidioides brasiliensis* yeasts were used to assess IL-1β, IL-18, TNF-α and IL-6 production by ELISA. Levels of IL-1β and IL-18 **(A,B)** and TNF-α and IL-6 **(C,D)** in the lungs of mice 48 h, 2, 4, and 10 weeks after infection with *P. brasiliensis* yeasts. Data represents one of two independent experiments using five mice per group (*n* = 5). Experiment was performed in triplicates represented as Mean ± SEM (**P < 0.01 and ***P < 0.001 compared with deficient mice).

The cytokines levels in lung homogenates of *WT, Nlrp3*^−/−^, *Casp1/11*^−/−^, *P2x7r*^−/−^, and *Asc*^−/−^ mice were analyzed at 48 h, 2, 4, and 10 weeks of *P. brasiliensis* infection. High levels of IL-1β and IL-18 secretion were observed in the lungs of *WT* mice at all postinfection periods studied, but a more robust production was observed at weeks 4 and 10 postinfection. Equivalent reduced levels of these cytokines were detected in the lung supernatants of *Nlrp3*^−/−^, *Casp1/11*^−/−^, *P2x7r*^−/−^, and *Asc*^−/−^ mice (Figures 3A,B). From 48 h up to 10 weeks of infection a modest increase of TNF-α and IL-6 secretion was observed in the lungs of all mouse strains studied, but no significant differences were observed at any period of infection (Figures 3C,D). These findings showed that in pulmonary PCM, TNF-α, and IL-6 production is independent on NLRP3 activation.

### NLRP3 Induces Increased Influx of PMN Leukocytes, into the Lungs of *P. brasiliensis* Infected Mice

**Figure 4:**
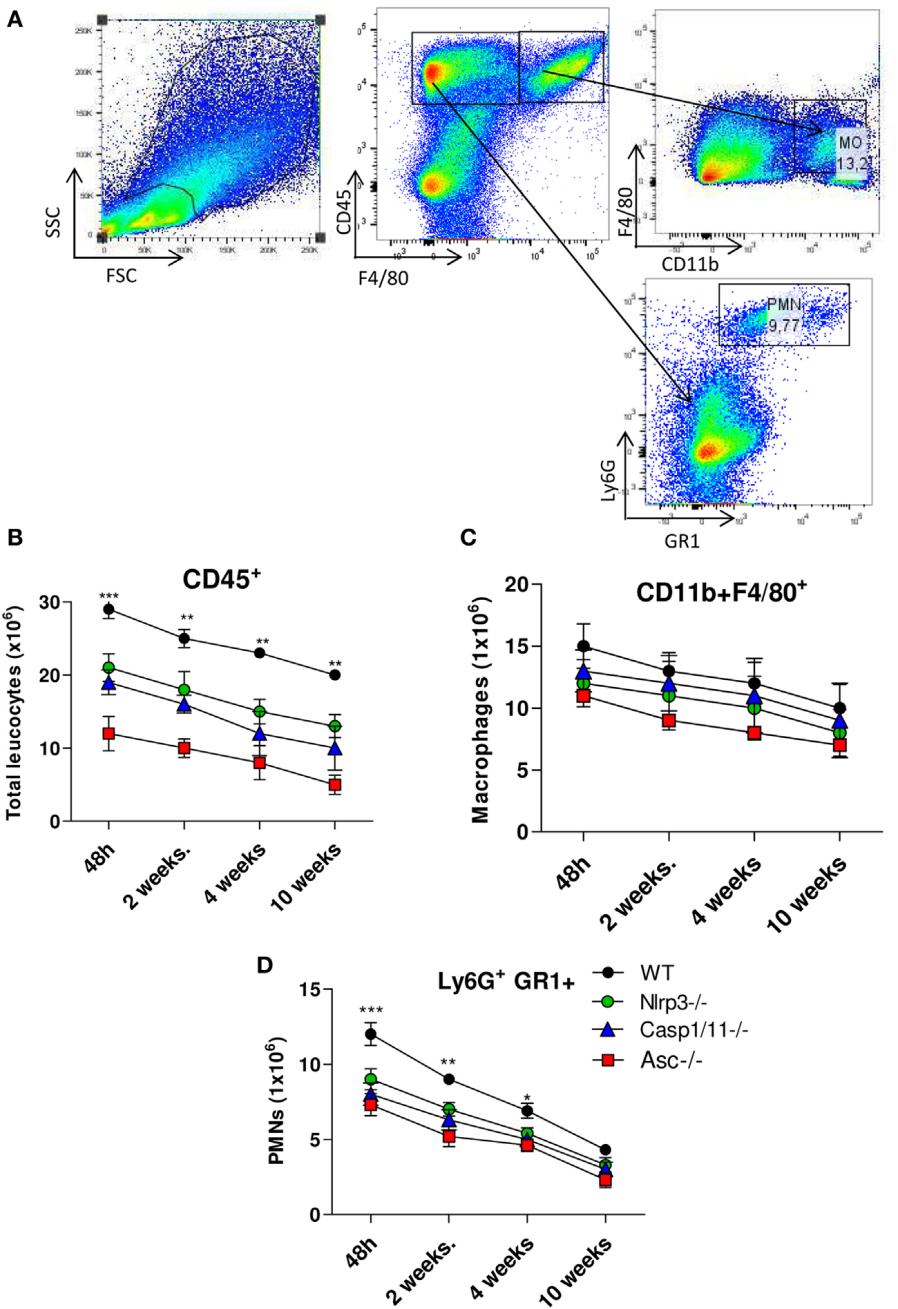
Activation of NLRP3 inflammasome induces increased influx of PMN leukocytes into the lungs of *Paracoccidioides brasiliensis* infected mice. Inflammatory cell infiltrates in the lungs of *WT, Nlrp3*^−/−^, *Casp1*^−/−^, and *Asc*^−/−^ mice infected with 1 × 10^6^ *P. brasiliensis* yeasts were analyzed at different postinfection periods (48 h, 2, 4, and 10 weeks) by flow cytometry. **(A)** Gates strategy using WT cells, **(B)** total leukocytes, **(C)** macrophages, and **(D)** PMN leukocytes were phenotyped by anti-CD45, F4/80, CD11b, GR1, and anti-Ly6G fluorochromes-labeled antibodies. Data represents one of two independent experiments using five mice per group (*n* = 5). Experiment was performed in triplicates represented as Mean ± SEM (*P < 0.05, **P < 0.01, and ***P < 0.001, compared with deficient mice).

The pulmonary cellular infiltrates in *WT, Nlrp3*^−/−^, *Casp1/11*^−/−^, and *Asc*^−/−^ mice were analyzed by flow cytometry at several periods of *P. brasiliensis* infection (Figure 4A). An increased influx of total leukocytes characterized as CD45^+^ cells was detected in the lungs of *WT* mice compared with *Nlrp3*^−/−^, *Casp1/11*^−/−^, and *Asc*^−/−^ mice, which was more expressive in the acute phase (48 h) of infection (Figure 4B). After 48 h and 2 weeks of infection, a high number of CD11b^+^F4/80^+^ macrophages were seen in the lungs of infected mice, that decreases at later periods, but no significant differences between *WT* and deficient mice were detected (Figure 4C). Interestingly, an increased influx of GR1^+^Ly6G^+^ PMN cells was seen in the lungs of *WT* mice in comparison with *Nlrp3*^−/−^, *Casp1/11*^−/−^, and *Asc*^−/−^ mice at 48 h, 2 and 4 weeks of infection (Figure 4D). These data showed that NLRP3 actively regulates the cell infiltration that occurs in the lungs of *P. brasiliensis* infected mice.

### NLRP3 Induces Increased Influx of Activated CD4^+^ and CD8^+^ T Cells into the Lungs of Infected Mice

**Figure 5:**
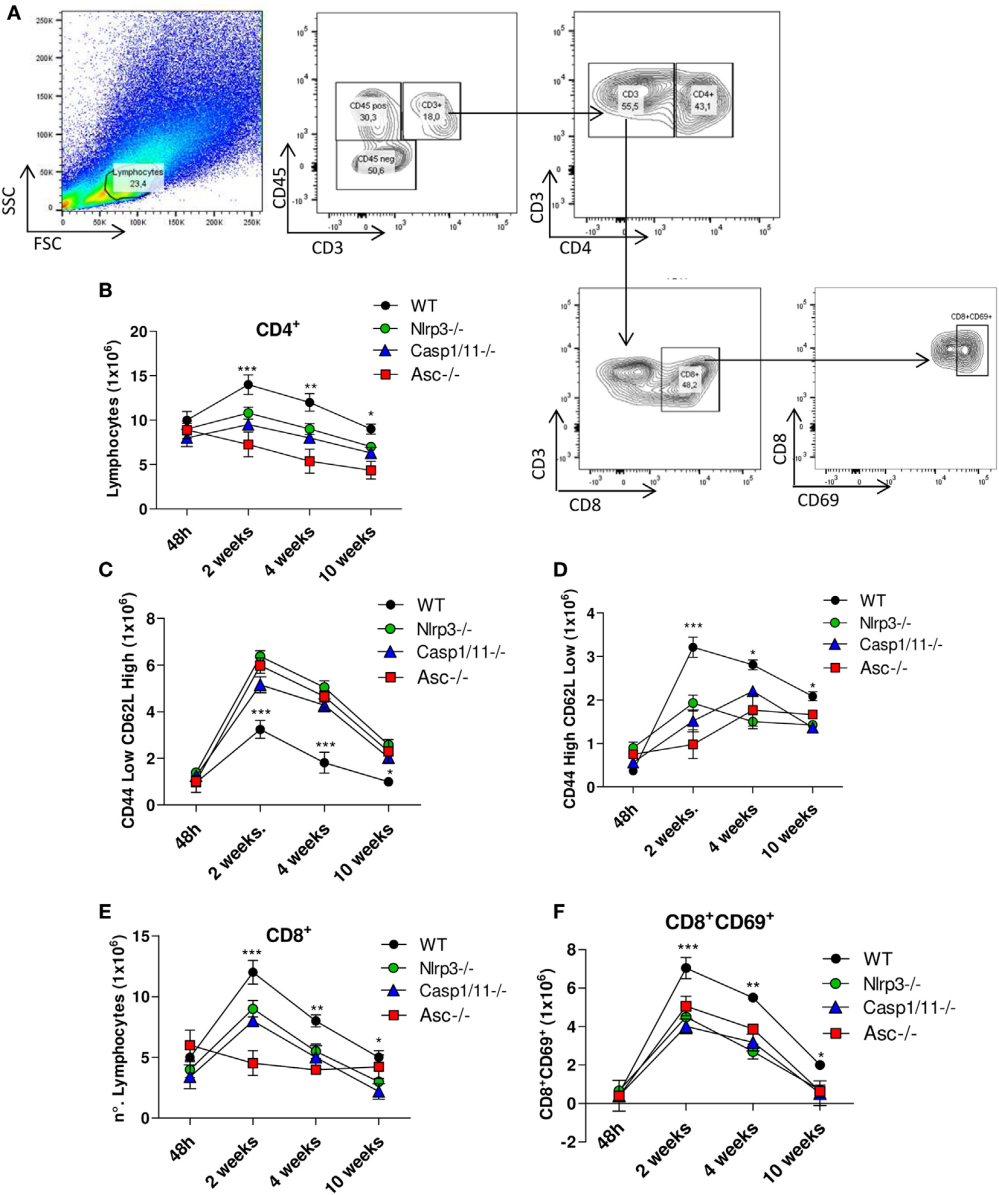
NOD-like receptor P3 (NLRP3) inflammasome induces the migration of activated CD4+ and CD8+ T cells into the lungs of *Paracoccidioides brasiliensis* infected mice. Inflammatory cell infiltrates in the lungs of *WT, Nlrp3*^−/−^, *Casp1*^−/−^, and *Asc*^−/−^ mice infected with 1 × 10^6^ *P. brasiliensis* yeasts were analyzed at different postinfection periods (48 h, 2, 4, and 10 weeks) by flow cytometry. **(A)** Gates strategy using lung leukocytes from WT mice. **(B)** CD4^+^ T cells were characterized using anti-CD4 labeled antibodies. **(C)** Naïve (CD44^low^CD62L^high^) and **(D)** memory/effector (CD44^high^CD62L^low^) CD4^+^ T lymphocytes were characterized using adequate gates and fluorochromes-labeled anti-CD4, CD44, and CD62L antibodies. **(E)** CD8^+^ and **(F)** CD8^+^CD69^+^-activated T cells were characterized using anti-CD8 and CD69 fluorochromes-labeled antibodies. Data represents one of two independent experiments using five mice per group (*n* = 5). Experiment was performed in triplicates represented as Mean ± SEM (*P < 0.05, **P < 0.01, and ***P < 0.001, compared with deficient mice).

The profile of CD4^+^ cell activation was characterized by flow cytometry (Figure 5A). From week 2 onward, CD4^+^ T lymphocytes appeared in increased numbers in the lungs of WT when compared with *Nlrp3*^−/−^, *Casp1/11*^−/−^, and *Asc*^−/−^ mice (Figure 5B). The subpopulations of naïve (CD4^+^CD44^low^CD62L^high^) and effector/memory (CD4^+^CD44^high^CD62L^low^) T cells were also determined. Our data showed increased numbers of naïve CD4^+^ T cells in the lungs of *Nlrp3*^−/−^, *Casp1/11*^−/−^, and *Asc*^−/−^ compared with WT mice at weeks 2, 4, and 10 of infection (Figure 5C). In contrast, WT mice presented increased numbers of pulmonary effector/memory CD4^+^ T cells at the same postinfection periods (Figure 5D). When the presence of activated CD8^+^ T cells was evaluated, an increased number of activated CD8^+^CD69^+^ T cells were detected in the lungs of WT mice at weeks 2, 4, and 10 of infection. In contrast, *Nlrp3*^−/−^, *Casp1/11*^−/−^, and *Asc*^−/−^ mice had diminished numbers of activated CD8+ CD9+ T cells at 2 and 4 weeks that were totally abolished at 10 weeks of infection (Figures 5E,F).

### NLRP3 Inhibits the Presence of Treg Cells into the Lungs of Infected Mice

**Figure 6:**
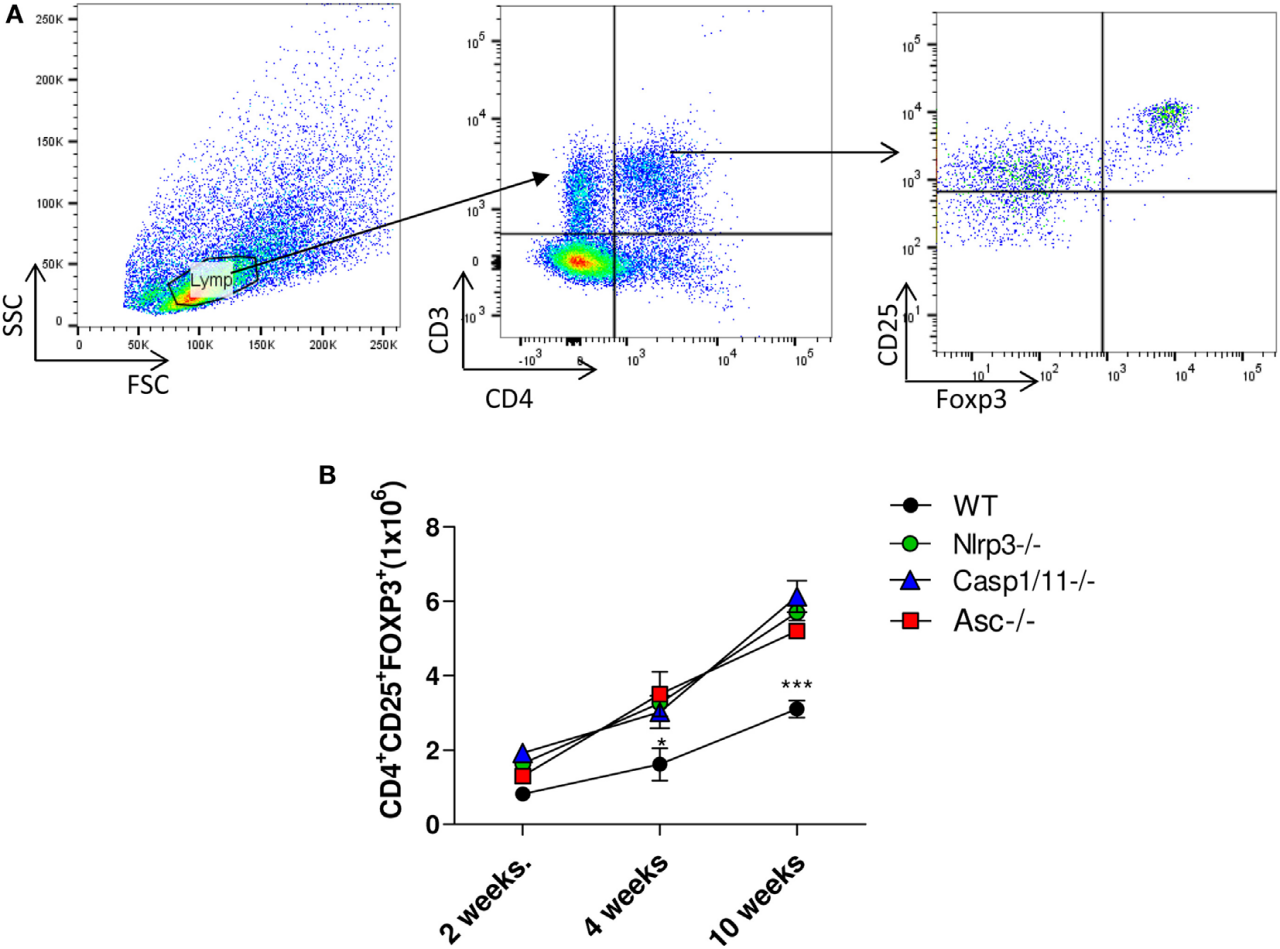
NOD-like receptor P3 (NLRP3) inflammasome inhibits the presence of regulatory T (Treg) cells in the lungs of infected mice. The number of Treg cells (CD4^+^CD25^+^FOXP3^+^) was determined in the lungs of *WT, Nlrp3*^−/−^, *Casp1/11*^−/−^, and *Asc*^−/−^ mice infected with 1 × 10^6^ *Paracoccidioides brasiliensis* yeasts at different postinfection periods (48 h, 2, 4, and 10 weeks) by flow cytometry. **(A)** Gates strategy using lung leukocytes from WT mice. **(B)** Treg cells (CD4^+^CD25^+^FOXP3^+^) were characterized using adequate gates and fluorochromes-labeled anti CD4, CD25, and FOXP3 antibodies. Data represents one of two independent experiments using five mice per group (*n* = 5). Experiment was performed in triplicates represented as Mean ± SEM (*P < 0.05, **P < 0.01, and ***P < 0.001, compared with deficient mice).

Regulatory T cells were characterized by the CD4^+^CD25^+^FOXP3^+^ phenotype as shown in the Figure 6A. An increased number of Treg cells was observed in the lungs of *Nlrp3*^−/−^, *Casp1/11*^−/−^, and *Asc*^−/−^ at 4 and 10 weeks of infection, compared with WT mice, which also showed increased number of these cells, although lower than NLRP3 components deficient mice (Figure 6B). These data showed that NLRP3 played a role in the control of adaptive immunity to *P. brasiliensis* infection by increasing the number of activated T cells in WT mice, as shown in Figure 5D, and reducing the presence of Treg cells that migrate to the lung of WT mice.

### NLRP3 Signaling Induces a Prevalent Expansion CD4^+^ and CD8^+^ T Cells Expressing IFN-γ and IL-17 with Concomitant Reduction of IL-4^+^ and TGF-β T Cells

**Figure 7:**
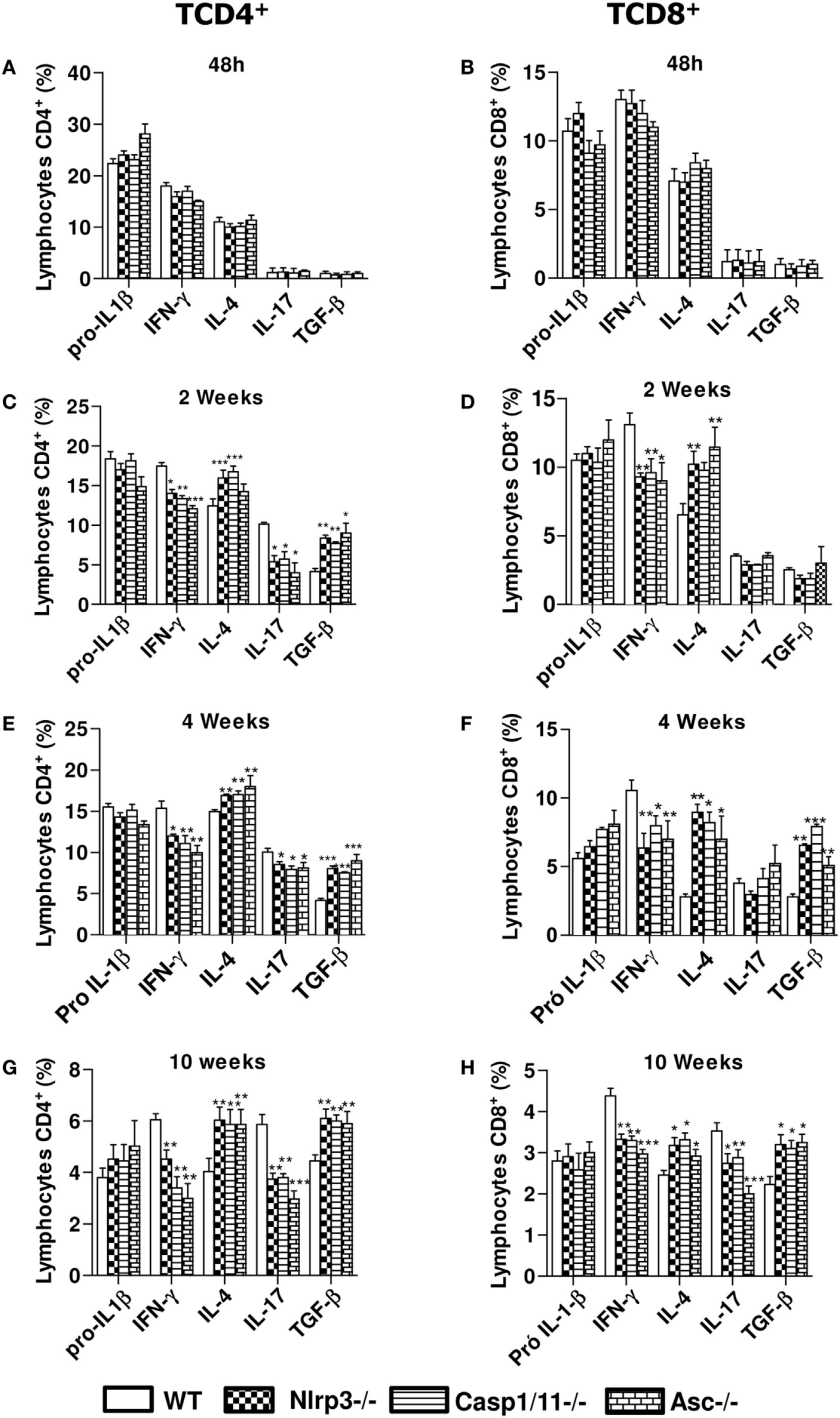
NOD-like receptor P3 (NLRP3) inflammasome induces a Th1/Th17 profile of immune response. Intracellular cytokine analysis from inflammatory infiltrates of lungs of *WT, Nlrp3*^−/−^, *Casp1/11*^−/−^, and *Asc*^−/−^ mice infected with 1 × 10^6^ *Paracoccidioides brasiliensis* yeasts was performed at 48 h **(A,E)**, 2 weeks **(B,F)**, 4 weeks **(C,G)**, and 10 weeks **(D,H)** by flow cytometry. CD4^+^ and CD8^+^ T cells were labeled with anti-CD4 and anti-CD8 antibodies, permeabilized and intracellular cytokines determined using fluorochromes-labeled anti- IL-1β, IFN-γ, IL-4, IL-17, and TGF-β antibodies. Data represents one of two independent experiments using five mice per group (*n* = 5). Experiment was performed in triplicates represented as Mean ± SEM (*P < 0.05, **P < 0.01, and ***P < 0.001, compared with WT mice).

We have further studied the expression of intracellular pro-IL-1β, IFN-γ, IL-4, IL-17, and TGF-β cytokines by CD4^+^ and CD8^+^ T cells in the lungs of infected *WT, Nlrp3*^−/−^, *Casp1/11*^−/−^, and *ASC*^−/−^ mice at 48 h, 2, 4, and 10 weeks of infection. Our data showed that in the acute phase of infection (48 h) there was an elevated frequency of CD4^+^ and CD8^+^ T cells expressing intracellular pro-IL-1β, IFN-γ, and IL-4 in the lungs of WT and deficient (*Nlrp3*^−/−^, *Casp1/11*^−/−^, and *ASC*^−/−^) mice, but no significant differences were observed between the mouse strains. At this period, no significant expression of IL-17 and TGF-β was detected in CD4^+^ and CD8^+^ T cells (Figures 7A,E). From 2 to 10 weeks of infection, increased frequencies of IFN-γ^+^ and IL-17^+^ CD4^+^ T cells were seen in the lungs of *WT* mice when compared with *Nlrp3*^−/−^, *Casp1/11*^−/−^, and *Asc*^−/−^ mice. In contrast, there was an increased percentage of IL-4^+^ and TGF-β^+^ CD4^+^ T cells in the lungs of *Nlrp3*^−/−^, *Casp1/11*^−/−^, and *Asc*^−/−^ compared with WT mice (Figures 7B–D). Generally, CD8^+^ T cells appeared in decreased frequencies when compared with CD4^+^ T cells, but a similar profile of cytokines expression was observed in both T cell subpopulations (Figures 7E–H). Interestingly, differences in IL-17 synthesis by CD8 T cell were only seen at week 10 postinfection. These data showed that NLRP3 played an important role in the polarization of type-1 and type-17 T cell responses in WT mice resulting in a better control of *P. brasiliensis* infection. In contrast, *Nlrp3*^−/−^, *Casp1/11*^−/−^, and *Asc*^−/−^ mice showed a prevalent Th2/Th3/Treg profile of response that was unable to control the fungal infection.

### *P. brasiliensis* Infected *Nlrp3*^−/−^, *Casp1/11*^−/−^, *P2x7r*^−/−^, and *Asc*^−/−^ Mice Show Increased Tissue Pathology and Decreased Survival Rates

**Figure 8:**
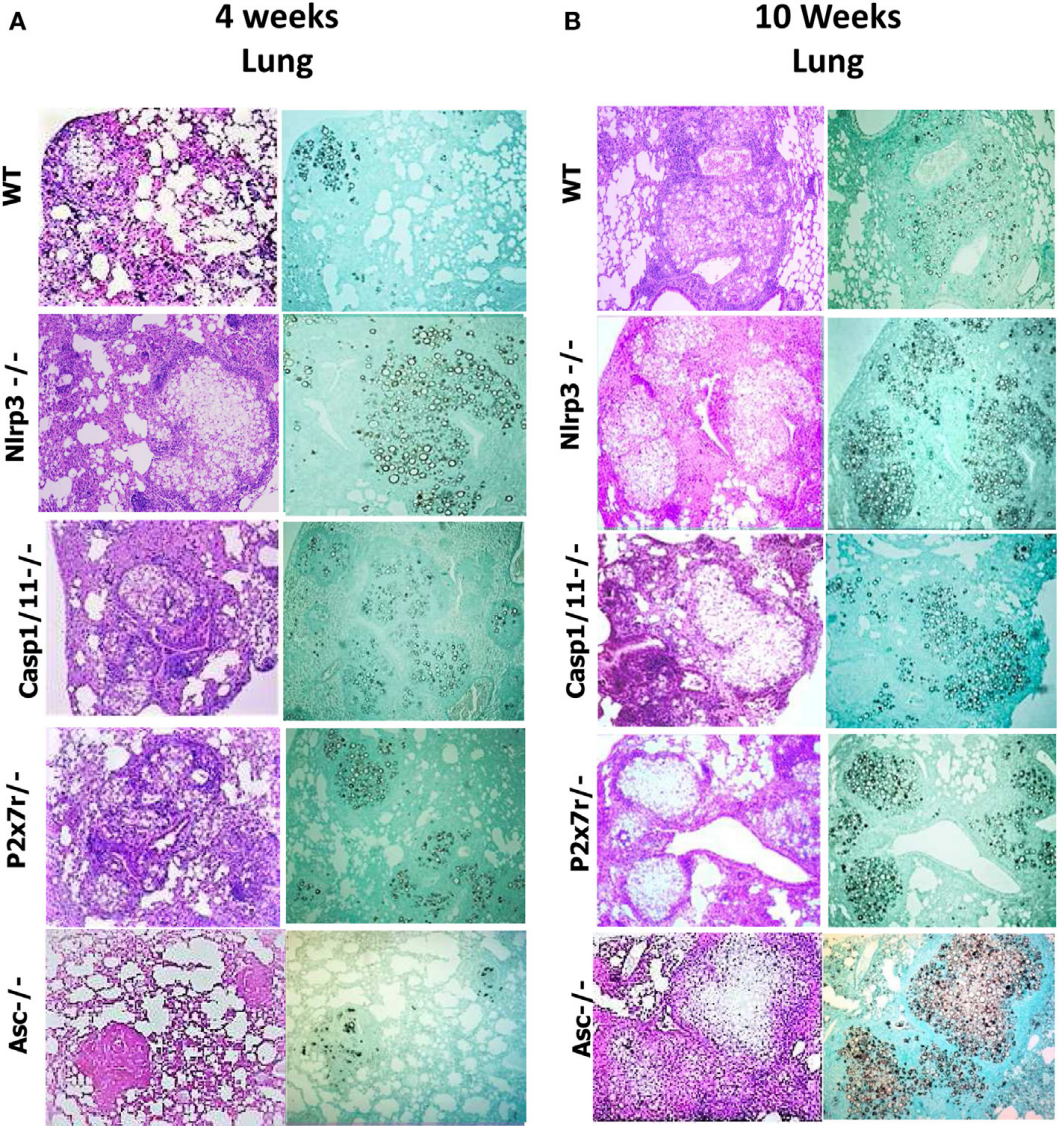
NOD-like receptor P3 (NLRP3) inflammasome activation leads to less severe lung pathology. Photomicrographs of lungs of *WT, Nlrp3*^−/−^, *Casp1/11*^−/−^, *P2x7r*^−/−^, and *Asc*^−/−^ mice infected i.t. with 1 × 10^6^ *Paracoccidioides brasiliensis* yeasts after 4 and 10 weeks of infection. **(A,B)** Histological lung sections were stained with hematoxylin–eosin [H&E, left panels of **(A,B)**] or Groccot [right panels of **(A,B)**].

To better clarify the importance of NLRP3 in the severity of *P. brasiliensis* infection, the histopathological analysis of the lung tissues of *WT* and *Nlrp3*^−/−^, *Casp1/11*^−/−^, *P2x7*^−/−^, and *Asc*^−/−^ mice was performed at weeks 4 and 10 of i.t. infection. The histological sections were stained with hematoxylin–eosin (H&E) for cellular identification and with Groccot stain for fungal identification and localization in the tissue (Figure 8). The degree of infection was evaluated based in the size and morphology of lung lesions as well as fungal presence and intensity of inflammatory infiltrates. At 4 weeks of infection, less extensive and severe pulmonary lesions were observed in the lungs of *WT* in comparison with *Nlrp3*^−/−^, *Casp1/11*^−/−^, *P2x7r*^−/−^, and *Asc*^−/−^ mice. The lungs of WT mice showed an inflammatory process that spread over the entire area of the organ. Large fungal loads were restricted to the center of lesions. The granulomas were large and coalescent, but there were some preserved organ areas. In *Nlrp3*^−/−^, *Casp1/11*^−/−^, *P2x7r*^−/−^, and *Asc*^−/−^ mice the extension and cellularity of the lesions as well as the presence of fungi were larger than in *WT* mice (Figure 8A). By 10 weeks of infection (Figure 8B), a similar pattern of lesions was detected, but at higher intensity. In comparison with week 4, a higher content of fungi within the granulomas and increased necrotic areas were seen at this postinfection period. In summary, the pathology developed by *Nlrp3*^−/−^, *Casp1/11*^−/−^, *P2x7r*^−/−^, and *Asc*^−/−^ mice was much more severe than that presented by *WT* mice corroborating the data of the increased fungal burden and impaired T cell immunity demonstrated by these mouse strains.

**Figure 9:**
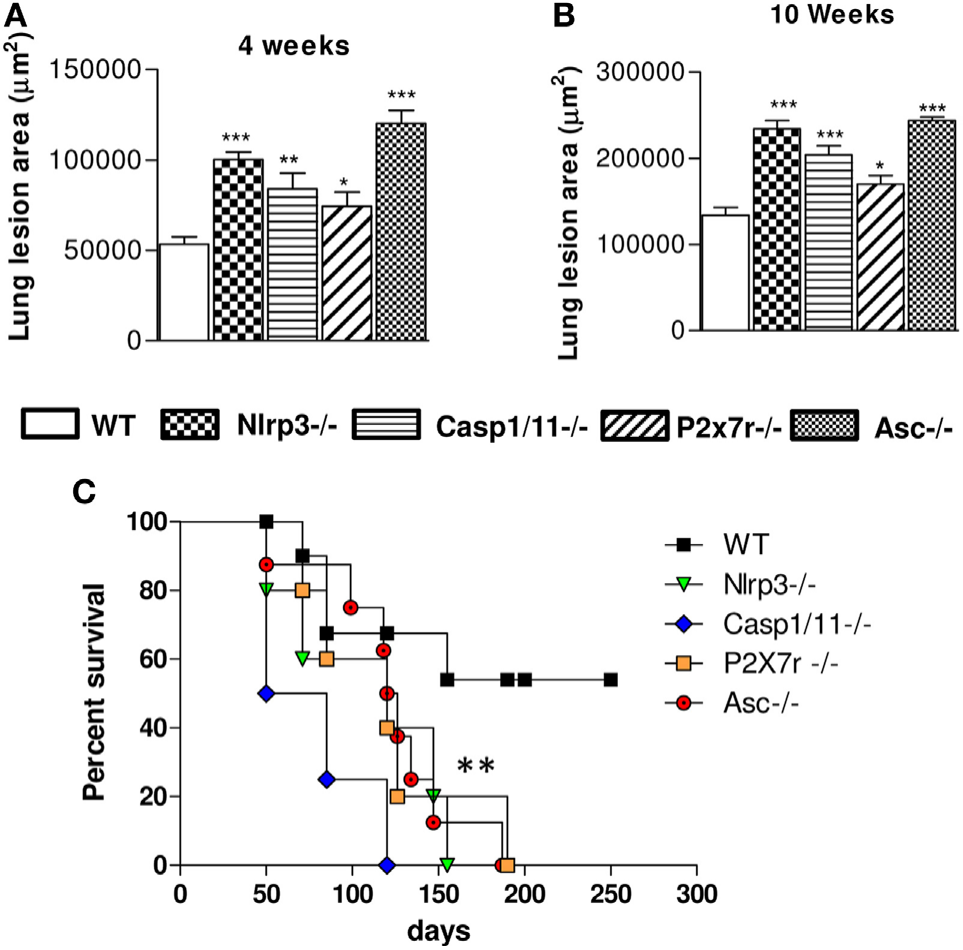
NOD-like receptor P3 (NLRP3) inflammasome activation determines decreased pulmonary pathology and increased survival rates of *Paracoccidioides brasiliensis* infected mice. The total areas of lung lesions were calculated in the periods of 4 **(A)** and 10 weeks **(B)** after infection. Data are expressed as the mean ± SEM of triplicates from two independent experiments. **(C)** The mortality studies were conducted with groups of 8 *WT, Nlrp3*^−/−^, *Casp1/11* ^−/−^, *Asc*^−/−^, and *P2x7r*^−/−^ mice i.t. infected with 1 × 10^6^ *P. brasiliensis*. Mice were monitored daily for a period of 250 days to determine the survival time (*P < 0.05, **P < 0.01, and ***P < 0.001, compared with WT mice).

The extent of lesions areas in the lungs (Figures 9A,B) reveals that mice deficient for NLRP3, Caspase-1/11, P2X7R, and ASC showed significantly larger lesions occupying higher tissue area than those present in WT animals. Mortality studies were done with groups of eight mice i.t. infected with one million *P. brasiliensis* yeasts. Infected mice were monitored daily for a period of 250 days to determine the survival time. As can be seen, *Nlrp3*^−/−^, *Casp1/11*^−/−^, *P2x7r*^−/−^, and *Asc*^−/−^ mice had a significantly shorter survival time than WT mice (Figure 9C), further confirming the crucial importance of NLRP3 in the immunoprotection of pulmonary PCM.

## Discussion

The role of NLRP3 inflammasome and the cooperation of TLRs and CLRs in the modulation of immune response against *P. brasiliensis* infection in macrophages and dendritic cells have been studied in our lab (8, 15, 18, 22). Using our murine model of resistance and susceptibility to PCM, it was verified that the activation of NLRP3 inflammasome by *P. brasiliensis* infection of macrophages from resistant A/J mice is dependent on dectin-1-Syk signaling (21). Other studies showed the role of TLR2, TLR4, dectin-1, and MyD88 signaling in the secretion of pro-inflammatory cytokines (IL-1β, TNF-α, and IL-6) and control of protective Th1/Th17 immune responses (5, 18, 20). Mice and patients infected with *P. brasiliensis* secrete IL-1β and IL-18, suggesting that NLRP3 is an important player in the recognition of this fungal pathogen (55, 56). The role of NLRP3 using *in vivo* models of PCM were less explored; however, a recent study using mice systemically infected with *P. brasiliensis* demonstrated that NLRP3 controls host resistance by inducing a prevalent Th1 immunity associated with IL-18 secretion (40). Because human PCM is acquired by the pulmonary route of infection, in the present study, we sought to characterize the role of NLRP3 inflammasome in i.t. infected mice whose response involves pulmonary-mediated immunological mechanisms. Several parameters of disease severity and host response were evaluated at several postinfection periods. Increased numbers of viable yeasts were recovered from lungs and livers of *Nlrp3*^−/−^, *Casp1/11*^−/−^, *P2x7r* ^−/−^, and *Asc*^−/−^ mice. Our CFU data corroborate with those of Ketelut-Carneiro et al. that found increased fungal loads in the lungs and livers of *Asc*^−/−^ and *Casp1/11*^−/−^ mice infected by the i.v. route (40). Our study has also revealed for the first time the importance of the purinergic P2X7 receptor (P2X7R) in NLRP3 activation by *P. brasiliensis* infection, in agreement with recent studies that demonstrated the participation of this receptor in the activation of the NLRP3 inflammasome and immunity against intracellular infections caused by *Mycobacterium, Chlamydia, Leishmania*, and *Toxoplasma* (57–60). In our pulmonary model of PCM, a marked influence of P2X7R in the immune response and disease severity of mice was clearly shown by the deficient fungicidal activity, decreased cytokines production, high tissue damage and decreased survival rates developed by P2X7R deficient mice. As expected, NLRP3 activation exerted a profound influence in the levels of IL-1β and IL-18 present in the lung tissue and subsequent polarization of immune response. This finding indicates that the new equilibrium in the pool of pulmonary cytokines were responsible by the diverse immunity developed by NLRP3 deficient mice, once the levels of other cytokines such as TNF-α and IL-6 remained in the control levels. It is well known that IL-1β and IL-18 contribute to the induction of the adaptive immune response by stimulation of T cell proliferation, differentiation of Th1/Th17 immunity and increased activity of NKT cytotoxic cells (61). NLRP3 and pro-IL-1β mRNAs are highly expressed in the first signal of NLRP3 activation, as a result of the engagement of PRRs and PAMPs on phagocytes (25, 62). Our findings demonstrated that the genetic deficiency of NLRP3, Casp1/11, and Asc totally impaired the expression of IL-1β, IL-18, NLRP3, Casp1, and ASC mRNA in the lungs of infected mice at week 4 of infection. Besides the control of NLRP3 inflammasome components, *P. brasiliensis* infection induced an increased expression of Syk, confirming our previous studies demonstrating the crucial role of dectin-1-Syk signaling in the activation of this receptor (21). It is well known that the definition of the inflammatory infiltrates that accompanies an infectious process helps to define the type and intensity of the established immune response. Here, the expression of NLRP3 inflammasome-associated components was concomitant with increased infiltration of PMNs but not macrophages into the lungs of mice, indicating the selective influence of NLRP3 activation on this important effector cell in fungal infections. PMN-rich inflammatory reactions characterize the function of Th17 cells that, besides the typical production of IL-17 and IL-22, are committed with the secretion of chemokines involved in the migration and activation of PMN leukocytes (8, 63–66). In agreement, an enhanced differentiation of Th17 and Tc17 cells were seen during the infection of NLRP3 sufficient mice. The NLRP3 inflammasome activation has also induced an increased influx and activation of CD4^+^ and CD8^+^ T cells to the lungs of mice that were probably involved in the control of fungal loads, and subsequent control of lung pathology. Indeed, decreased numbers of CD4^+^- and CD8^+^-activated T cells were consistently seen in the lungs of *Nlrp3*^−/−^, *Casp1/11*^−/−^, and *Asc*^−/−^ mice that developed increased fungal burdens associated with increased lung pathology and mortality rates. In pulmonary PCM, both T cell subsets participate in the protective mechanisms, with an intense and prominent contribution of CD8^+^ T cells (67, 68). Although in less number, CD8^+^ T cells were here shown to participate in the immunity associated with NLRP3 activation, a relevant observation added to the role of this receptor in the host defense mechanisms against *P. brasiliensis* infection.

The caspase-1-dependent cytokines exert important effects in the initiation of the adaptive Th1 and Th17 cellular responses to fungal infections (37). IL-1β has been suggested to promote inflammatory diseases by inducing the expansion of differentiated T cells (69) and has been implicated, alone or in combination with IL-6 and TGF-β, in driving murine and human Th17 priming and phenotype stabilization (66, 69). IL-18 was initially discovered as an IFN-γ-inducing factor, which together with IL-12 promotes the development of Th1 cells (70). Here, we assessed the intracellular cytokines production to define the effect of NLRP3 activation in the Th1, Th2, and Th17 defense mechanisms of *P. brasileinsis* infected mice. A new balance of Th cells subsets was seen in the absence of NLRP3 inflammasome components: an increased expression of IL-4 and TGF-β that accompanied a reduced number of IFN-γ^+^ and IL-17^+^ CD4^+^ and CD8^+^ T cells. In murine PCM, we previously demonstrated that IL-4 can exert protective or deleterious roles, depending on the mouse strain studied (71). It can dampen excessive inflammatory reactions of B10.A mice but suppresses the protective Th1 immunity of *C57BL/6* mice. A recent report showed that IL-4 suppresses NLRP3-dependent caspase-1activation and subsequent IL-1β secretion (72). These data show a mutual control between NLRP3 inflammasome and IL-4, as here observed. Our study also showed a clear association between NLRP3 activation and decreased synthesis of TGF-β and Treg cells development suggesting that the predominant pro-inflammatory milieu produced by this receptor significantly reduced the regulatory anti-inflammatory mechanisms. Interestingly, in a model of chronic kidney disease, the expression of NLRP3 was linked to the control of TGF-β-dependent signaling (73) although in our model enhanced TGF-β expressing cells were allied with absence of NLRP3 activation.

The analysis of intracellular cytokines revealed that NLRP3 played an important role in the induction of Th1 and Th17 responses with concomitant reduction of Th2/Treg expansion, a process of immune regulation associated with regressive disease in both humans and experimental models of PCM (2, 3, 5–8, 10). In addition, an important contribution of CD8^+^ T cells to IL-17 and IFN-γ production was also seen. Although no studies have directly addressed the role of IL-17 in PCM, several indirect findings clearly demonstrated that a well-balanced production of pro- and anti-inflammatory cytokines is crucial to determine a protective immunity. The evaluation of cytokines production by patients with polar forms of PCM has shown that the prevalent synthesis of Th1/Th17 cytokines characterized the mild forms of chronic PCM, whereas an elevated production of IL-4 and IL-9, associated with high numbers of Treg cells, were linked with the severe forms of the disease (10). Our previous studies have also demonstrated that a balanced differentiation of Th1/Th17/Treg cells is fundamental to achieve immunoprotection in pulmonary PCM. Indeed, the absence of TLR2 signaling induces excessive pathology associated with increased Th17 differentiation an impaired Treg development (6). In contrast, in TLR4 deficiency the excessive proliferation of Treg cells and reduced Th17 differentiation led to more severe disease (7). Importantly, the absence of dectin-1 signaling induced a critical reduction in all IL-17 secreting cells, particularly IL-17^+^CD8^+^ T cells and a severe PCM (20). These findings, and those here reported clearly indicate that the equilibrium in the expansion of T cell subpopulations is fundamental to immunoprotection against PCM.

The findings here reported are in contrast with those of Ketelut-Carneiro et al. showing that an enhanced Th1 immunity controlled by increase production of IL-18 was responsible by the protective role of NLRP3 inflammasome in a murine model of PCM (40). These discrepancies, however, could be attributed to the route of infection used. Indeed, the study of Ketelut-Carneiro et al. used i.v. infected mice (40), whereas our studies were done with i.t. infected animals. In this aspect, our previous studies have clearly shown the influence of the route of infection in the severity of the disease and immune response of *P. brasiliensis* infected mice (41). We verified that the s.c. route induces a self-healing infection in several mouse strains allied with elevated delayed hypersensitivity reactions. Interestingly, the previous s.c. infection of B10.A mice led to immunoprotection or disease exacerbation depending on the route of fungal challenge. Immunoprotection was achieved after intraperitoneal challenge and was associated with persistent cell-mediated immunity and a mixed type-1/type-2 immunity. Exacerbated disease was found after intravenous challenge and was linked with anergy of cellular immunity and prevalent type-2 immune response (41). In contrast to the prevalent Th1/Th17 immunity induced by NLRP3 inflammasome activation in *WT* mice, *Nlrp3*^−/−^, *Casp1/11*^−/−^, and *Asc*^−/−^ mice showed increase expansion and migration of Treg cells to the site of infection that possibly have contributed to the deficient control of fungal loads and non-protective inflammatory reactions. In agreement, the survival studies showed increased mortality rates among *Nlrp3*^−/−^, *Casp1/11*^−/−^, *Asc*^−/−^, and *P2x7r*^−/−^ mice accompanying increased fungal burdens and areas of lung lesions. In conclusion, the more efficient immunity mediated by Th1 and Th17 cells associated with NLRP3 inflammasome activation is highly protective to pulmonary PCM and this intracellular receptor appears to be significantly involved in the control of signaling pathways that led to the integration of innate and adaptive host immune responses to *P. brasiliensis* infection.

## Ethics Statement

Animal experiments were performed in strict accordance with the Brazilian Federal Law 11,794 establishing procedures for the scientific use of animals and the State Law establishing the Animal Protection Code of the State of São Paulo. All efforts were made to minimize suffering, and all animal procedures were approved by the Ethics Committee on Animal Experiments of the Institute of Biomedical Sciences of University of São Paulo (Proc.76/04/CEUA).

## Author Contributions

Conceived and designed experiments: VC and CF. Contributed with reagent: DZ. Performed the experiments: CF, TC, EA, FL, and NG. Analyzed the data: CF, VC, FL, EA, and DZ. Wrote the paper: CF and VC.

## Conflict of Interest Statement

The authors declare that the research was conducted in the absence of any commercial or financial relationships that could be construed as a potential conflict of interest.
